# Beyond Conventional Methods: Rapid and Precise Quantification of Polyphenols in *Vigna umbellata* via Hyperspectral Imaging Enhanced by Multi-Scale Residual CNN

**DOI:** 10.3390/s26082356

**Published:** 2026-04-11

**Authors:** Hao Liang, Xin Yang, Nan Wang, Xinyue Lu, Wenwu Zou, Aicun Zhou, Xiongwei Lou, Yufei Lin

**Affiliations:** 1 College of Mathematics and Computer Science, Zhejiang A&F University, Hangzhou 311300, China; lhao@zafu.edu.cn (H.L.); 2023611032035@stu.zafu.edu.cn (X.Y.); nan@zafu.edu.cn (N.W.); 2024611031022@stu.zafu.edu.cn (X.L.); 2 Institute of Modern Agriculture and Health Care Industry, Wencheng 325300, China; 13968830330@163.com; 3 National Key Laboratory for Development and Utilization of Forest Food Resources, Zhejiang A&F University, Hangzhou 311300, China; aczhou@zafu.edu.cn; 4 Wenzhou Key Laboratory of AI Agents for Agriculture, Wenzhou Academy of Agricultural Sciences, Wenzhou 325006, China

**Keywords:** *Vigna umbellata*, polyphenol content, hyperspectral imaging, residual convolutional neural network, nondestructive detection

## Abstract

*Vigna umbellate*, a typical edible and medicinal crop, is rich in polyphenolic compounds with antioxidant, antibacterial, anti-inflammatory, and lipid-regulating activities. However, traditional methods for polyphenol content detection rely on chemical analysis, which is cumbersome and time-consuming, making it difficult to meet the demands of high-throughput rapid detection. Although hyperspectral imaging technology offers the potential for non-destructive and rapid detection, existing analytical methods are often limited by issues such as high spectral band redundancy, insufficient feature extraction, and inadequate model stability, which constrain prediction accuracy and practical application potential. To address this, this study proposes a multi-scale residual convolutional neural network (MS-RCNN) based on competitive adaptive reweighted sampling (CARS) for feature band selection, combined with near-infrared hyperspectral imaging technology, to construct a rapid and non-destructive prediction model for the polyphenol content of *Vigna umbellata*. The model employs a parallel multi-scale convolutional module to extract spectral features with different receptive fields, and incorporates residual connections and adaptive pooling mechanisms to enhance feature reuse and robustness. Experiments compared the performance of partial least squares regression (PLSR), least squares support vector machine (LS-SVM), multi-scale convolutional neural network (MS-CNN), and MS-RCNN models. The results indicate that the MS-RCNN model based on CARS screening achieved the best prediction performance, with a coefficient of determination (R^2^) of 0.9467, a root mean square error of prediction (RMSEP) of 0.0448, and a residual predictive deviation (RPD) of 4.33. Compared with the optimal PLSR and LSSVM models, its R^2^ values were improved by 0.2078 and 0.1119, respectively. In summary, the MS-RCNN model proposed in this study enables rapid, non-destructive, and accurate prediction of polyphenol content in *Vigna umbellata*, providing an efficient technical approach for quality detection of edible and medicinal crops.

## 1. Introduction

Adzuki bean refers to the dried mature seeds of *Vigna umbellata* (Chixiaodou) and *Vigna angularis* (Chidou), which are annual herbaceous plants belonging to the legume family. Given the superior medicinal quality of the former, this study employs *Vigna umbellata* as the experimental material. It was first documented in the *Shennong Bencao Jing (Divine Farmer’s Materia Medica)* and classified as a “medium-grade” medicinal substance [1], with subsequent extensive records appearing in various historical material medica and medical texts throughout later dynasties. This herb is traditionally recognized for its effects in promoting diuresis to reduce edema, as well as detoxifying and draining pus. *Vigna umbellata* has been progressively better understood for its role in promoting human health and exemplifying the concept of “medicinal and edible homology [2],” driven by advances in detection technology and deeper medical research. The polyphenols in *Vigna umbellata* have antioxidant properties, which can effectively eliminate oxygen free radicals in the human body, prevent cell aging, and prevent cancer [3], enhance human immunity and prevent the occurrence of diseases such as inflammation, cancer, atherosclerosis, and diabetes. At the same time, polyphenols have a good therapeutic effect on light-induced retinal damage [4,5]. Furthermore, these polyphenols can improve blood circulation, thereby reducing the incidence of cardiovascular and cerebrovascular diseases and lowering cholesterol levels [6]. Therefore, studying the polyphenol content in *Vigna umbellata* holds significant theoretical and practical importance for evaluating its medicinal efficacy, pharmacological effects, and pharmacological activity. Currently, common methods for measuring polyphenols include the Folin–Ciocalteu method [7], liquid chromatography [8], and spectrophotometry [9], among others. These methods, as industry standard practices, have high detection accuracy. However, these techniques often involve complex procedures, require relatively expensive equipment, intricate reagent preparation, and time-consuming detection processes, rendering them unsuitable for large-scale rapid screening. Therefore, developing a non-destructive, efficient, and rapid detection method is of significant importance for ensuring the quality of *Vigna umbellata*.

Hyperspectral imaging (HSI) integrates spectroscopic detection and computer vision technologies into a single system, enabling the acquisition of both spectral and spatial information from target objects without causing damage to the samples [10]. By integrating spatial and spectral data from hundreds of continuous bands, it has become a transformative tool in the field of non-invasive chemical analysis [11]. Its applications span agricultural quality assessment [12], environmental monitoring [13], and pharmaceutical research, and are gradually extending into the field of traditional Chinese medicine for the quantitative analysis and quality control of bioactive ingredients [14]. Yu et al. [15] described the richness of information in hyperspectral data based on the application of HSI in detecting active ingredients, assessing their quality, and identifying the authenticity and variety of traditional Chinese medicine, and emphasized the importance of texture features in the analysis. hyperspectral data inherently contain rich spectral fingerprint information while retaining subtle spatial distribution features. This dual characteristic not only increases computational complexity but also affects analytical performance due to strong inter-band correlations and informational overlap between adjacent spectral bands [16].

To overcome these limitations, researchers have introduced deep learning techniques into the field of hyperspectral analysis [17]. Deep learning can learn and extract knowledge from vast amounts of data to accomplish complex tasks [18,19]. In recent years, the integration of Convolutional Neural Networks (CNN), a representative deep learning algorithm, with HSI spectroscopy has attracted significant attention from researchers. CNN are a class of feedforward neural networks that incorporate convolutional operations. Through their multi-layer architecture, they are capable of extracting deep-level features from spectral data, demonstrating strong feature extraction capabilities and high model expressiveness [20,21]. Furthermore, CNN operate as end-to-end models, requiring minimal or even no data preprocessing, thereby reducing operational complexity [22]. Consequently, the application of CNNs in HSI modeling demonstrates strong practical potential and research value. As one of the most advanced techniques in agricultural quality monitoring, CNN have achieved superior performance in certain chemometrics tasks compared to classical methods such as Partial Least Squares (PLS) and Support Vector Machines (SVM) [23,24]. Gu et al. [25] developed a 1D CNN model based on HSI for non-destructive prediction of polysaccharide and flavonoids content in Anoectochilus roxburghii and found that the model outperformed the traditional SVM model. Wang et al. [26] combined HSI with a deep learning-based Temporal Convolutional Network-Attention (TCNA) model, enabling simultaneous and effective prediction of six rare ginsenosides in ginseng. However, as the depth of convolutional layers and the dimensionality of channels in CNNs continue to increase, optimization may stagnate due to gradient decay during training, which can hinder stable learning of deep-level features [27]. Residual modules introduce shortcut connections with identity mapping, providing a pathway for gradients to propagate across layers. This effectively mitigates the vanishing gradient problem in deep networks and promotes more stable and efficient model training [28].

Addressing the above limitations, this study innovatively proposes a MS-RCNN, whose core innovations are reflected in two aspects. First, a parallel multi-scale convolutional module is designed, employing one-dimensional convolutional kernels of different sizes to extract spectral features in parallel, enabling the model to simultaneously capture short-range, medium-range, and long-range spectral dependencies, thereby achieving cross-scale extraction and fusion of spectral features related to polyphenol content. Second, a residual connection structure is embedded after the multi-scale feature concatenation, providing a shortcut for gradient propagation through cross-layer identity mapping, which alleviates gradient vanishing while enhancing feature reuse, thus improving the stability of model training and prediction accuracy. This synergistic design overcomes the limitations of traditional CNN, such as fixed receptive fields and the tendency for deep networks to degrade, offering a novel architectural approach for nonlinear modeling in hyperspectral analysis.

The objective of this study is to achieve rapid and non-destructive assessment of polyphenol content in the medicinal material *Vigna umbellata*. A convolutional neural network with residual connections (RCNN) combined with HSI is proposed for the determination of polyphenol content in *Vigna umbellata*. The specific objectives of this study are: (1) to select the optimal PLSR and LSSVM models for predicting polyphenol content in *Vigna umbellata* through different preprocessing methods and effective wavelength selection techniques; (2) to develop and evaluate a deep learning framework for determining polyphenol content in *Vigna umbellata*; (3) to investigate whether residual modules can enhance the performance of CNN in polyphenol prediction; and (4) to compare the performance of the four methods: PLSR, LSSVM, CNN, and RCNN.

## 2. Materials and Methods

### 2.1. Preparation of Experimental Samples

All experimental samples were procured from specialized traditional Chinese medicine markets across multiple provinces in China. In accordance with the quality control standards of the Chinese Pharmacopoeia, a total of 191 qualified *Vigna umbellata* samples were purchased from provinces including Yunnan, Henan, Hebei, Guangxi, and Guizhou, Among them, 42 samples were from Yunnan, 38 from Henan, 35 from Hebei, 38 from Guangxi, and 38 from Guizhou. All samples underwent quality screening, including origin identification and morphological characterization, conducted by professional technicians at the Quality Inspection Center of the Traditional Chinese Medicine Research Institute in Pan’an County, Jinhua City, Zhejiang Province. All samples were stored in a refrigerator at −20 °C and equilibrated at room temperature for 24 h prior to spectral acquisition to eliminate temperature effects. Among these samples, seven-tenths were randomly assigned as the calibration set, while the remaining three-tenths were used as the prediction set.

### 2.2. Measurement of Polyphenol Content in Vigna umbellata

After acquiring hyperspectral images of the *Vigna umbellata* samples, the samples were pretreated by drying them to constant weight and then grinding them into a uniform powder. Accurately 0.4 g of *Vigna umbellata* powder sample was weighed, placed in a centrifuge tube, and 25 mL of 70% ethanol solution was added, mixed well, and allowed to stand for 15 min. Subsequently, ultrasonic extraction was performed at a power of 500 W, a frequency of 40 Hz, and a temperature of 40 °C for 1 h. After extraction, the sample was removed and allowed to cool to room temperature. The cooled sample solution was placed in a centrifuge and centrifuged at 8000 rpm for 5 min. The supernatant was collected for later use. Accurately 400 μL of the supernatant was pipetted into a 10 mL volumetric flask, 3 mL of ultrapure water was added and mixed well; then 1.5 mL of 10% Folin–Ciocalteu reagent was added, shaken well, 4 mL of 8% sodium carbonate (Na_2_CO_3_) solution was added, and finally the mixture was diluted to the mark with ultrapure water, mixed well, and allowed to stand in the dark for 15 min to complete the colorimetric reaction. After color development, 200 μL of each test solution was added to a 96-well plate, and each sample was spotted three times. The absorbance of each solution was measured at a wavelength of 765 nm [29]. In this experiment, the Folin–Ciocalteu method [30] was used to determine the total polyphenol content of *Vigna umbellata*. Gallic acid was used as the reference standard to establish the calibration curve. The regression equation was Y = 3.9755x + 0.0519 with a correlation coefficient R^2^ = 0.9995, showing excellent linearity. Each sample was measured in triplicate, and the average value was used as the reference value for the polyphenol content to reduce random measurement errors. The statistical results of polyphenol content are shown in Table 1.

### 2.3. Hyperspectral Image Acquisition and Correction

The hyperspectral image system consists of a near-infrared hyperspectral imager (GaiaField-N17E, Dualix Spectral Imaging, Sichuan Shuangli Hepu Technology Co., Ltd, Chengdu, China), indoor test chamber (HSIA-BD), four halogen lamps (50 W), lifting table, computer and supporting software (Optiplex 7080MT/SpecView). The near-infrared hyperspectral imager has a spectral range of 900–1700 nm, spatial resolution of 640 pixels, bands of 512 and spectral resolution of 5 nm. The size and lifting range of lifting table are 300 × 300 mm and 90–370 mm, respectively. To obtain hyperspectral images, before measurement, we spread the *Vigna umbellata* samples were placed in a black circular Petri dish with a height of 1 cm and a radius of 3 cm, and pressed down to ensure uniform sample thickness. After repeated adjustments and optimizations, the distance between the lens and the sample was adjusted to 40 cm, the platform moving speed was 5 mm/s, and the camera exposure time was 7 ms. Each sample was measured three times. Then, the original hyperspectral image $I_{O}$ was calculated for black and white corrected. The acquired original image data reflects the signal intensity, and the reflectance of the spectrum needs to be calculated by black and white correction. Blank calibration was performed using a white calibration plate to set the maximum emissivity (~99%), and dark correction was performed by covering the lens cap to set the minimum reflectance (~0%) [31]. The corrected image was calculated using the following formula:

(1) $$I=\frac{I_{O}-B}{W-B}$$

In the formula, $I_{O}$ is the original hyperspectral image, W is the whiteboard calibration image, and B is the blackboard calibration image.

### 2.4. Spectral Extraction and Preprocessing

**Figure 1:**
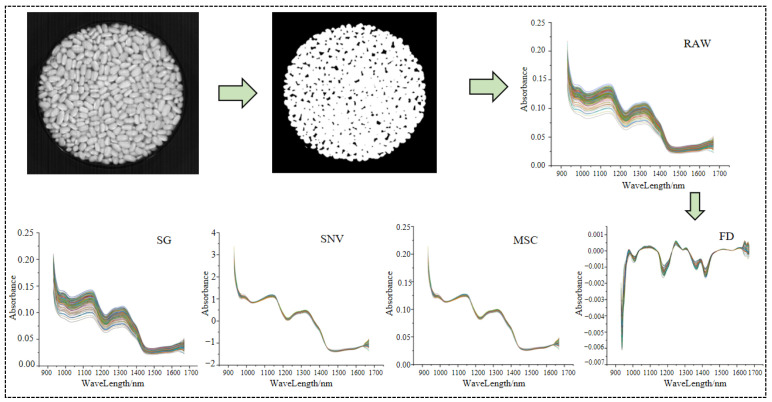
Hyperspectral image segmentation and spectral extraction results.

To eliminate the interference of the black background on subsequent spectral feature extraction, a single-band guided adaptive background segmentation strategy was adopted. First, the 1290 nm band image was extracted from the hyperspectral data cube, and the global optimal threshold was adaptively determined via the Otsu’s method to achieve pixel-level separation between *Vigna umbellata* samples and the background. Then, a binary mask was generated, where the target regions were labeled as valid pixels (1) and the background as invalid pixels (0). Finally, the mask was applied band-by-band to the entire hyperspectral data cube, and the black background of all bands was stripped by pixel masking operation, thus accurately extracting the region of interest (ROI) for each sample. To improve the prediction accuracy and reproducibility, the reflectance values of *Vigna umbellata* in each hyperspectral image were averaged to obtain the mean spectrum [32]. Subsequently, the mean spectra extracted from three hyperspectral images of the same *Vigna umbellata* sample scanned in parallel were averaged again, which was used as the final spectral data for the sample, as shown in Figure 1.

Given the severe distortion of spectral data at the start and end of the spectral range, 472 bands within 940–1670 nm were retained in this study. Even so, the complete elimination of other noises in the original spectra could not be guaranteed; thus, spectral preprocessing was performed, and the spectral curves after preprocessing are shown in Figure 1. Spectral preprocessing is capable of eliminating spectral noises such as high-frequency random noise and baseline drift, thereby improving the model performance [33,34]. In this study, four preprocessing methods were applied to the original spectra of samples, including multiplicative scatter correction (MSC), Savitzky–Golay smoothing (SG), first derivative (FD), and standard normal variate transformation (SNV) [35,36,37,38]. The optimal preprocessing method was ultimately selected based on the performance of the full-spectrum model.

### 2.5. Selection of Effective Wavelengths

Hyperspectral data are characterized by high dimensionality and multicollinearity, making them difficult and time-consuming to process. To improve computational speed and achieve real-time detection, variable selection methods are needed to select significant wavelengths and reduce the dimensionality of hyperspectral data [39,40]. This study uses Competitive Adaptive Reweighted Sampling (CARS) and Sequence Projection Algorithm (SPA) to select significant wavelengths in polyphenols from *Vigna umbellata*.

CARS is an effective wavelength selection method. Its core idea is to simulate the natural law of “survival of the fittest” and select the feature wavelengths most relevant to the prediction target from high-dimensional spectral data through an iterative competition mechanism. This method combines Monte Carlo random sampling with variable importance analysis of partial least squares (PLS) models [41,42]. First, a Monte Carlo strategy is used to perform multiple random subset samplings. Second, an exponentially decreasing function is used to forcibly reduce the number of wavelengths, achieving coarse screening. Next, an adaptive reweighted sampling mechanism is adopted to competitively retain and eliminate wavelengths based on the absolute value of the regression coefficients of each wavelength in the PLS model, allowing important wavelengths to accumulate weight. Finally, cross-validation is used to evaluate the wavelength subsets generated in each iteration, and the optimal combination of feature wavelengths is determined based on the minimum root mean square error of cross-validation. In the CARS algorithm, wavelengths with low absolute values of regression coefficients are gradually eliminated during the iteration process, while key wavelengths are retained because of their significant contribution to the prediction model, thereby improving the interpretability and prediction accuracy of the model while reducing dimensionality.

SPA (Successive Projections Algorithm) is a feature selection algorithm for signal processing and numerical optimization [43]. The principle is to iteratively project the data into a low-dimensional subspace and identify the variables that contribute most significantly to distinguishing different categories or groups in the data. This method is based on vector projection analysis, which selects the combination of feature variables with the lowest information redundancy from spectral data. In the SPA analysis, multiple linear regression (MLR) is used to build predictive models for different subsets of variables, and the root mean square error (RMSE) of each model is calculated. Finally, the subset of variables with the smallest RMSE is determined as the optimal wavelength combination.

### 2.6. Establishment and Assessment of Models

#### 2.6.1. Traditional Machine Learning Modeling Methods

Partial least squares regression (PLSR) is an efficient and interpretable multivariate statistical technique that extracts a series of latent variables that maximize the covariance of the independent variables (spectral matrix) and the dependent variable (target value) matrix by simultaneously decomposing them. While effectively overcoming the problem of multicollinearity in high-dimensional data, a robust linear regression model is established. Its core lies in determining the optimal number of latent variables through cross-validation to avoid overfitting [44,45].

Least Squares Support Vector Machine (LSSVM) is an efficient regression algorithm based on the support vector machine framework [46,47]. By introducing a least-squares loss function, the inequality-constrained optimization problem in traditional support vector machines is transformed into a convex optimization problem of solving a system of linear equations, thereby significantly reducing computational complexity. This model not only inherits the powerful advantages of support vector machines in processing small sample, nonlinear and high-dimensional data, but also has excellent anti-overfitting ability and generalization performance due to the optimized design of its regularization parameters and kernel function parameters.

#### 2.6.2. Deep Learning Modeling Methods

**Figure 2:**
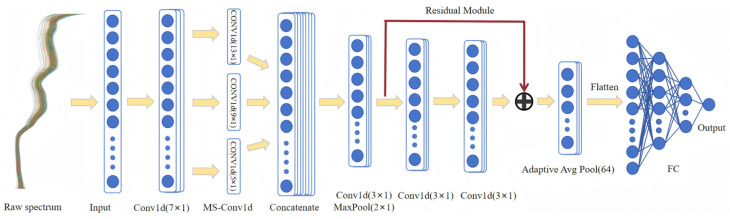
MS-RCNN model structure diagram.

Convolutional Neural Networks (CNN) have become the mainstream deep learning method for processing high-dimensional spectral data due to their powerful feature learning and pattern recognition capabilities [48]. Its basic structure typically includes convolutional layers, pooling layers, and fully connected layers, which can automatically extract complex features from data. However, traditional CNN often suffer from the following key problems when dealing with complex spectral signals with high dimensions and high noise: First, the receptive field of a single-scale convolutional kernel is fixed, making it difficult to capture the local and global correlations of spectral data at different wavelength dimensions [49]; second, deep networks are prone to gradient vanishing and network degradation, leading to reduced model training efficiency [50]. Therefore, this study proposes a multi-scale residual convolutional neural network (MS-RCNN), as shown in Figure 2. This model incorporates residual connections into the classic CNN architecture and constructs a parallel multi-scale convolution module. It aims to alleviate gradient vanishing and network degradation through cross-layer identity mapping. At the same time, it designs a parallel multi-scale convolution module, using three different sizes of one-dimensional convolution kernels: 13 × 1, 9 × 1, and 5 × 1, to capture long-range, mid-range, and short-range spectral features respectively, thereby expanding the model’s receptive field coverage of spectral signals. After the feature fusion layer completes the convolutional dimensionality reduction and pooling operations, it is connected to a residual module containing two 3 × 1 convolutional layers to further enhance the feature representation. Then, adaptive average pooling is used to unify the feature sequence length to 64 dimensions, and finally, the predicted value is output through three fully connected layers.

#### 2.6.3. Model Evaluation

The performance of the model is evaluated using several key metrics: coefficient of determination (R^2^), root mean square error (RMSE), and relative percentage deviation (RPD). An ideal model is characterized by low RMSE, high R^2^, and high RPD. An RPD value in the range of 2.5–3 indicates good prediction accuracy, and a value greater than 3 is considered a good value for quantitative prediction [51]. The calculation methods for these evaluation parameters are as follows:

(2) $$R^{2}=1-\frac{\sum_{j=1}^{n}{\left(X_{j}-Y_{j}\right)}^{2}}{\sum_{j=1}^{n}{\left(X_{j}-\bar{X}\right)}^{2}}$$

(3) $$RMSE=\sqrt{\frac{\sum_{j=1}^{n}{\left(X_{j}-Y_{j}\right)}^{2}}{n}}$$

(4) $$RPD=\frac{SD}{RMSE}$$

where $X_{j}$ and $Y_{j}$ represent the true value and predicted value of sample j, respectively, $\bar{X}$ represents the mean of the true values, n is the sample size, and SD is the standard deviation.

## 3. Results and Discussion

### 3.1. Spectral Analysis

**Figure 3:**
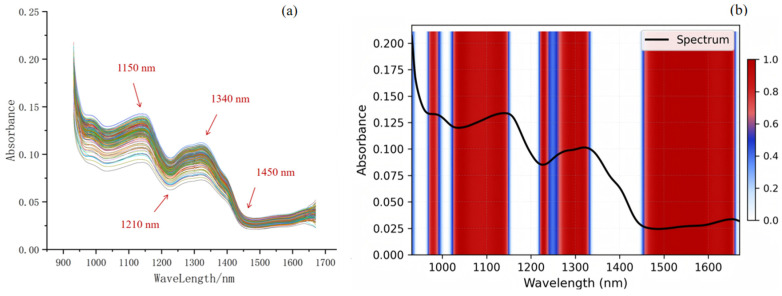
(**a**) Original spectrum; (**b**) Grad-CAM Heatmap from the MS-RCNN Model.

Figure 3a shows the original average spectral reflectance curves of all *Vigna umbellata* samples. Although there is some overlap and intersection in the spectra of different samples, their overall trends are similar. Several distinct characteristic peaks and bands are visible in the spectrum, mainly around 1150, 1210, 1340 and 1450 nm. The peaks around 1150 nm and 1210 nm can be attributed to the influence of the second harmonic of the stretching vibration of C-O and C-H bonds [52,53]; the absorption around 1340 nm may be due to the second harmonic of C-H bond stretching [54], while the peak around 1450 nm may be caused by the first harmonic of O-H and C-H bond stretching in water and carbohydrates [52]. Because polyphenols have a basic carbon skeleton of benzene ring (C_6_), their molecules contain a variety of characteristic chemical bonds such as C-H, O-H, and C-O [55]. Therefore, based on the vibrational spectral information of these chemical bonds, hyperspectral imaging technology can be used to predict the polyphenol content of a sample. Before modeling, in order to eliminate noise interference, the 900–940 nm and 1670–1700 nm band data with low signal-to-noise ratios were discarded. Finally, a total of 472 spectral variables in the 940–1670 nm range were used for modeling.

### 3.2. Prediction Analysis Based on Full Wavelengths

**Figure 4:**
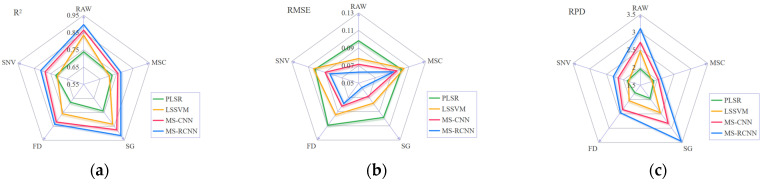
Model Performance Comparison: (**a**) R^2^ Performance Comparison of Four Models Under Four Pretreatment Methods; (**b**) RMSE Performance Comparison of Four Models Under Four Pretreatment Methods; (**c**) RPD Performance Comparison of Four Models Under Four Pretreatment Methods.

Based on full-wavelength hyperspectral data, this study systematically constructed four prediction models, including partial least squares regression (PLSR), least squares support vector machine (LSSVM), multi-scale convolutional neural network (MS-CNN), and multi-scale residual convolutional neural network (MS-RCNN), and compared their predictive performance. For these four models, this study further explored the effects of four spectral preprocessing methods (MSC, SG, FD, and SNV) on the models’ prediction results. It should be noted that there is no unified standard for the selection of spectral preprocessing methods, and their effectiveness is highly dependent on data characteristics and modeling objectives. Appropriate spectral preprocessing can effectively eliminate noise in hyperspectral data, correct scattering effects, and enhance characteristic information related to the research object; in contrast, improper preprocessing may introduce unnecessary errors into the data or damage effective characteristic information, thereby affecting the final modeling effect. Among them, MSC eliminates the influence of differences in the physical state of the sample by correcting multiplicative scattering [35]; SG uses local polynomial fitting to retain spectral morphology features while denoising [36]; SNV eliminates multiplicative scattering by standardizing each spectrum individually [37]; and FD eliminates baseline drift and enhances absorption peak features by calculating the spectral slope [38]. The results for each model are shown in Table 2. The results demonstrate that the deep learning models comprehensively outperform traditional methods in terms of accuracy. MS-CNN, with SG preprocessing, achieves an R^2^ of 0.8750, significantly outperforming PLSR (0.7396) and LSSVM (0.8347). Figure 4 clearly shows that the original spectral data, after SG preprocessing, exhibits high R^2^, low RPD, and low RMSE. Furthermore, it can be observed that the MS-RCNN model, with the further introduction of residual structures, achieves optimal performance under the same conditions, with an R^2^ of 0.9161 and an RPD as high as 3.45. This not only quantifies the performance gain brought by residual connections but also verifies the crucial role of this structure in enhancing the feature extraction and fusion capabilities of deep networks.

Figure 3b shows the Grad-CAM visualization heatmap based on the MS-RCNN model. The red areas represent the spectral bands with the highest attention the model showed when predicting the polyphenol content of *Vigna umbellata*, while the white areas represent the lowest attention. For near-infrared hyperspectral data, the model significantly focused on multiple bands at 1100 nm, 1300 nm, and 1450 nm and beyond. Notably, these high-attention regions precisely cover the three main peaks of the spectral curve, encompassing not only the characteristic bands related to polyphenolic compounds described in Section 3.1 but also identifying several additional discriminative spectral intervals. This phenomenon indicates that the MS-RCNN model can comprehensively and accurately capture the key information features of the spectral curve and effectively extract deep spectral patterns related to the target chemical components. This result further validates the effectiveness and reliability of the MS-RCNN model in hyperspectral data feature extraction and regression prediction tasks.

### 3.3. Prediction Analysis Based on Effective Wavelengths

Compared to PLSR and LSSVM models, CNN models exhibit superior feature learning capabilities. To improve model accuracy and uncover key spectral information, this study introduces CARS and SPA to select effective wavelengths across the entire spectrum. These two methods aim to automatically identify and filter the feature wavelengths most relevant to the polyphenol content of *Vigna umbellata*, thereby eliminating redundant information and reducing data dimensionality. This reduces computational complexity while further enhancing the model’s prediction accuracy and generalization ability. In the full-wavelength-based prediction analysis, the results show that the prediction accuracy of the spectral data after SG preprocessing and the original spectral data is stable and higher than that of other preprocessed spectral data in each model. Therefore, the original spectrum and the spectral data after SG preprocessing were screened respectively, and the extracted wavelength variables are shown in Table 3.

The effects of different wavelength selection methods on PLSR, LSSVM, MS-CNN, and MS-RCNN models were compared. The results showed that the performance of deep learning models was significantly improved after CARS selection, but the performance decreased after SPA selection. However, the performance of traditional machine learning models improved after SG preprocessing. The R^2^ and RPD of PLSR increased from 0.7396 and 1.96 in the full band to 0.8632 and 2.70, respectively, while the R^2^ and RPD of LSSVM increased from 0.8347 and 2.47 to 0.8364 and 2.47.

To address the performance degradation of deep learning models after SPA wavelength selection, this study analyzes the phenomenon from the following two aspects. First, the SPA is based on a linear assumption and aims to select variable combinations with minimal collinearity. In contrast, deep learning models rely on the continuity and integrity of input information. The wavelength subset selected by SPA disrupts the continuous structure of the spectral curve, thereby weakening the ability of CNN to extract local features. Second, the characteristic wavelengths selected by SPA exhibit a distribution pattern characterized by “concentration at both ends and sparsity in the middle” (as shown in Table 3). Among the 30 wavelengths selected by RAW + SPA, 14 were concentrated at the beginning and 5 at the end. SG + SPA exhibited a similar pattern, with multiple key intervals closely related to polyphenols (such as 1150–1450 nm) being largely eliminated, resulting in excessive compression of critical spectral information. In contrast, the characteristic wavelengths selected by CARS were more concentrated in the peak and valley regions of the spectral curve, better preserving the continuous morphology of the spectrum, and thus demonstrating better synergy with deep learning models.

As shown in Table 4. However, the R^2^ and RPD of the MS-CNN model after CARS processing increased from 0.8750 and 2.83 in the full band to 0.9199 and 3.61, respectively, while the R^2^ and RPD of MS-RCNN increased from 0.9161 and 3.45 to 0.9474 and 4.36, respectively, with all indicators reaching the optimal level. It is worth emphasizing that even on data with optimized features, MS-RCNN maintains a stable advantage over MS-CNN. This result further confirms that the performance gain brought by the residual structure is an inherent architectural advantage of the model, independent of the input features.

**Figure 5:**
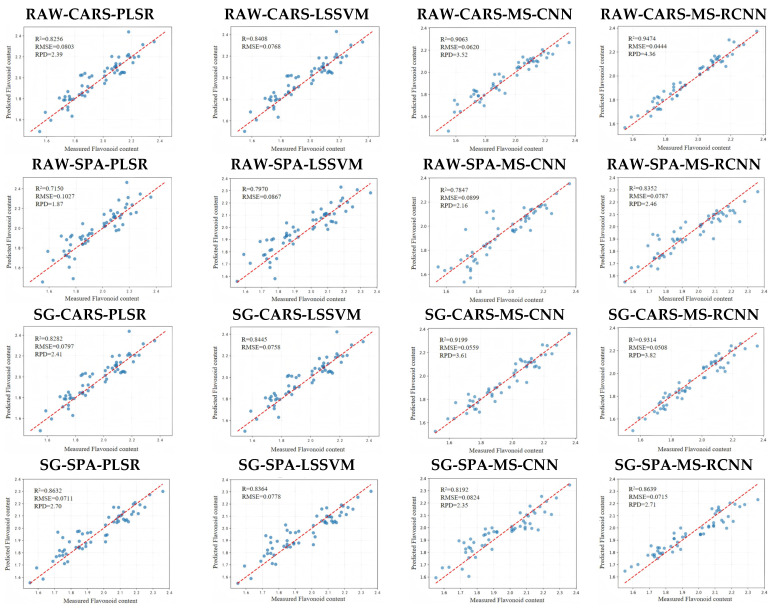
Scatter plot of prediction results from different models based on different effective wavelength selection methods.

The prediction results based on effective wavelengths from different models show that the deep learning model outperforms the traditional machine learning model in predicting the polyphenol content of *Vigna umbellata*. Figure 5 shows a scatter plot of the measured and predicted total polyphenol content of *Vigna umbellata* samples based on different models. Overall, the MS-RCNN model after CARS screening has the highest prediction accuracy, with the scatter plots distributed on both sides of the dashed line y = x, and an R^2^ of 0.9474. The proposed MS-RCNN model is an effective tool for achieving high-precision prediction of *Vigna umbellata* polyphenol content.

## 4. Discussion

This study aims to rapidly and non-destructively predict the polyphenol content of *Vigna umbellata.* A multi-scale residual convolutional neural network (MS-RCNN) was constructed and its predictive performance was systematically explored by combining it with near-infrared hyperspectral imaging technology. At the same time, the auxiliary effects of PLSR, LSSVM, MS-CNN and other models, as well as four preprocessing methods, CARS, and SPA feature band screening were compared and verified. It’s clear that deep learning models comprehensively surpass traditional machine learning methods in prediction accuracy. The core difference stems from the fundamentally different feature extraction mechanisms: traditional models rely on manual feature design and linear assumptions, making it difficult to fully capture the complex nonlinear correlations in the spectrum, with an optimal R^2^ of only 0.8364, while deep learning models achieve automatic feature extraction and hierarchical combination through convolutional layers, significantly improving expressive power. MS-RCNN, through a collaborative design of multi-scale convolutions and residual connections, captures multi-range correlated spectral features using convolutional kernels of different sizes, while mitigating gradient vanishing and enhancing cross-layer feature reuse through residual connections. Its feature extraction and fusion capabilities are significantly superior to MS-CNN without residual structures, achieving an R^2^ of 0.9199 for full-band modeling with SG preprocessing. Further combining this with CARS feature band filtering to remove redundant information further improves the R^2^ to 0.9474 for the original spectrum.

The collaborative design of multi-scale convolutional modules and residual connections in the MS-RCNN model overcomes the limitations of insufficient feature extraction capabilities and easy degradation of deep networks in traditional models, enabling it to more efficiently mine complex nonlinear information related to polyphenol content in hyperspectral data. This technical solution not only overcomes the drawbacks of traditional chemical detection methods, such as time consumption and sample damaging, but also provides a new model architecture for non-destructive detection of agricultural product components.

## 5. Conclusions

This study integrates competitive adaptive reweighted sampling feature selection, multi-scale residual convolutional neural network, and near-infrared hyperspectral imaging technology to achieve rapid, non-destructive, and high-precision prediction of *Vigna umbellata* polyphenol content. The main conclusions are as follows: In terms of model architecture, MS-RCNN, through the collaborative design of multi-scale convolution and residual connections, significantly outperforms PLSR, LSSVM models and MS-CNN without residual structure in feature extraction and fusion capabilities. It effectively breaks through the limitations of traditional models in processing complex hyperspectral data and verifies the core advantages of this architecture in hyperspectral nonlinear modeling. In terms of data optimization, CARS screens feature bands directly related to the structure of polyphenols from a chemometric perspective, providing the model with more chemically targeted and high-quality input. Unprocessed spectral data achieved optimal prediction results under the MS-RCNN model, with an R^2^ of 0.9474 and an RPD of 4.36. All performance indicators significantly outperform traditional methods, demonstrating good practicality and reliability.

This study still has certain limitations. Although the sample sources covered multiple provinces, the effects of factors such as variety, growing environment, harvest time, and storage conditions on polyphenol content were not systematically considered. Therefore, the generalizability of the model across different origins and batches requires further validation. In addition, this study was conducted on a single variety of *Vigna umbellata*, and whether the proposed model architecture can be effectively extended to the detection of active components in other edible and medicinal crops (such as Coix seed, Cassia seed, etc.) remains to be investigated in future research. Based on the above limitations, future studies will further expand sample sources to include adzuki bean samples from more origins, varieties, and different growth conditions to systematically evaluate the robustness and generalizability of the model. Meanwhile, the MS-RCNN model framework will be transferred to the detection of active components in other edible and medicinal crops to verify its applicability and transferability across different substrate types.

The MS-RCNN model constructed in this study demonstrates excellent practical value and reliability. It not only overcomes the limitations of traditional chemical analysis methods, such as time consumption and sample loss, and provides an efficient and accurate technical means for the quality evaluation of *Vigna umbellata*, but also provides a core model design reference for the rapid and non-destructive detection of active ingredients in other food and medicinal crops with innovative architecture, thus promoting the in-depth application of hyperspectral detection technology in the field of agricultural product quality evaluation.

## Figures and Tables

**Table 1:** Statistical analysis of polyphenol content in *Vigna umbellata*.

Index	Range (%)	Mean (%)	Variance (%^2^)
polyphenol	0.30~0.69	0.49	0.037

**Table 2:** Results of PLSR, LS-SVM, MS-CNN, and MS-RCNN models.

Model	Pretreatment Methods	R^2^	RMSE	RPD
PLSR	RAW	0.7390	0.0983	1.96
	MSC	0.7231	0.1012	1.90
	SG	0.7396	0.0982	1.96
	FD	0.6783	0.1091	1.76
	SNV	0.7196	0.1018	1.89
LSSVM	RAW	0.8355	0.0780	2.47
	MSC	0.7063	0.1042	1.85
	SG	0.8347	0.0782	2.46
	FD	0.7597	0.0943	2.04
	SNV	0.7093	0.1037	1.85
MS-CNN	RAW	0.8639	0.0715	2.71
	MSC	0.7570	0.0956	2.03
	SG	0.8750	0.0685	2.83
	FD	0.8192	0.0824	2.35
	SNV	0.7847	0.0899	2.16
MS-RCNN	RAW	0.8957	0.0626	3.10
	MSC	0.7755	0.0918	2.11
	SG	0.9161	0.0562	3.45
	FD	0.8352	0.0787	2.46
	SNV	0.8106	0.0844	2.30

**Table 3:** Characteristic Wavelength Information.

Method	All-Band Number	Number of Characteristic Variables	Specific Band
RAW + CARS	472	48	931.22, 1130.51, 1132.08, 1139.93, 1141.50, 1143.07, 1144.64, 1199.56, 1201.13, 1202.70, 1204.27, 1205.84, 1207.41, 1208.98, 1210.55, 1212.12, 1213.69, 1215.25, 1216.82, 1218.39, 1219.96, 1221.53, 1223.10, 1340.80, 1342.37, 1343.94, 1345.51, 1347.07, 1348.64, 1350.21, 1351.78, 1353.35, 1354.92, 1356.49, 1358.06, 1359.63, 1361.20, 1362.77, 1364.34, 1433.38, 1434.95, 1436.52, 1438.09, 1439.66, 1441.23, 1442.80, 1444.37, 1445.94
SG + CARS	472	47	931.22, 932.78, 1141.50, 1143.07, 1144.64, 1146.21, 1199.56, 1201.13, 1202.70, 1204.27, 1205.84, 1207.41, 1208.98, 1210.55, 1212.12, 1213.69, 1215.25, 1216.82, 1218.39, 1219.96, 1221.53, 1223.10, 1342.37, 1343.94, 1345.51, 1347.07, 1348.64, 1350.21, 1351.78, 1353.35, 1354.92, 1356.49, 1358.06, 1359.63, 1361.20, 1362.77, 1364.34, 1433.38, 1434.95, 1436.52, 1438.09, 1439.66, 1441.23, 1442.80, 1444.37, 1445.94, 1447.51
RAW + SPA	472	30	931.22, 932.78, 934.35, 935.92, 937.49, 939.06, 940.63, 942.20, 943.77, 945.34, 946.91, 948.48, 950.05, 951.62, 981.43, 983.00, 984.57, 986.14, 987.71, 989.28, 990.85, 992.42, 993.99, 1536.96, 1649.94, 1651.51, 1653.08, 1654.65, 1656.22, 1670.34
SG + SPA	472	41	931.22, 932.78, 934.35, 935.92, 937.49, 939.06, 1180.73, 1372.18, 1373.75, 1375.32, 1376.89, 1378.46, 1380.03, 1381.60, 1383.17, 1384.74, 1386.31, 1387.87, 1389.44, 1391.01, 1392.58, 1394.15, 1395.72, 1397.29, 1419.26, 1420.83, 1422.40, 1423.97, 1425.54, 1427.11, 1428.68, 1430.25, 1431.81, 1433.38, 1434.95, 1436.52,1438.09, 1439.66, 1441.23, 1442.80, 1670.34

**Table 4:** Results of polyphenol content prediction using different modeling and feature selection methods.

Method	Model	R^2^	RMSE	RPD
RAW + CARS	PLSR	0.8256	0.0803	2.39
	LSSVM	0.8408	0.0768	2.51
	MS-CNN	0.9163	0.0602	3.52
	MS-RCNN	0.9474	0.0444	4.36
RAW + SPA	PLSR	0.7150	0.1027	1.87
	LSSVM	0.7970	0.0867	2.22
	MS-CNN	0.7847	0.0899	2.16
	MS-RCNN	0.8352	0.0787	2.46
SG + CARS	PLSR	0.8282	0.0797	2.41
	LS-SVM	0.8445	0.0758	2.54
	MS-CNN	0.9199	0.0559	3.61
	MS-RCNN	0.9314	0.0508	3.82
SG + SPA	PLSR	0.8632	0.0711	2.70
	LSSVM	0.8364	0.0778	2.47
	MS-CNN	0.8192	0.0824	2.35
	MS-RCNN	0.8639	0.0715	2.71

## Data Availability

The data presented in this study are available on request from the corresponding authors.
